# Pseudomalignancies in Children: Histological Clues, and Pitfalls to Be Avoided

**DOI:** 10.3390/dermatopathology8030042

**Published:** 2021-08-14

**Authors:** Sébastien Menzinger, Sylvie Fraitag

**Affiliations:** 1 Department of Dermatology, University Hospital of Geneva, 1205 Geneva, Switzerland; 2 Department of Clinical Pathology, University Hospital of Geneva, 1205 Geneva, Switzerland; 3 Department of Pathology, Hôpital Necker-Enfants Malades, APHP, 75015 Paris, France; sylvie.fraitag@aphp.fr

**Keywords:** pseudomalignancies, pediatrics, pseudolymphoma, pityriasis lichenoides, lymphomatoid papulosis, mycosis fungoides, lymphoplasmacytic plaque, acral pseudo-lymphomatous angiokeratoma of children, histiocytic infiltrate, langerhans cell histiocytosis, CD1a+ dendritic cell hyperplasia, melanocytic disorders, congenital melanocytic lesion, proliferative nodule, Spitz tumor

## Abstract

The term “pseudomalignancy” covers a large, heterogenous group of diseases characterized by a benign cellular proliferation, hyperplasia, or infiltrate that resembles a true malignancy clinically or histologically. Here, we (i) provide a non-exhaustive review of several inflammatory skin diseases and benign skin proliferations that can mimic a malignant neoplasm in children, (ii) give pathologists some helpful clues to guide their diagnosis, and (iii) highlight pitfalls to be avoided. The observation of clinical–pathological correlations is often important in this situation and can sometimes be the only means (along with careful monitoring of the disease’s clinical course) of reaching a firm diagnosis.

## 1. Introduction

The term “pseudomalignancy” covers a large, heterogenous group of diseases characterized by a benign cellular proliferation, hyperplasia, or infiltrate that resembles a true malignancy clinically or histologically. Several inflammatory skin diseases or benign skin proliferations in children can mimic malignant neoplasms [1]. However, when examining a child’s skin biopsy, the pathologist must bear in mind that benign disorders are more frequent than malignancies.

Here, we review cellular infiltrates of the skin that can mimic neoplasms of the skin in children. The article is divided into three subsections, according to the specific type of cellular infiltrate (lymphocytic, histiocytic, and melanocytic infiltrates).

## 2. Lymphocytic Infiltrates

Cutaneous lymphocytic infiltrates that mimic lymphoma can be referred to cutaneous pseudo-lymphomas. This heterogenous group has been described in the literature as reactive lymphoproliferation that histopathologically and clinically imitates cutaneous lymphomas [2]. The causes are many and varied. Cutaneous lymphocytic infiltrates can be subdivided as a function of the histological pattern or the predominant immunophenotype (T or B), amongst others.

### 2.1. Vitiligo

Many inflammatory skin disorders can mimic mycosis fungoides (MF) clinically or histologically; this is the case for vitiligo. This acquired chronic depigmentation disorder results from the selective destruction of melanocytes; although the etiology is unknown, an auto-inflammatory or auto-immune mechanism is most strongly suspected [3].

Clinical differentiation between vitiligo and MF in children can be challenging, especially because hypopigmented MF is particularly frequent in children with a darker skin type (Fitzpatrick types IV–VI) [4] and can clinically mimic vitiligo or other pathologies with hypopigmentation or depigmentation (e.g., pityriasis alba, pityriasis versicolor, post-inflammatory hypopigmentation, progressive macular hypomelanosis, and hypopigmented pityriasis lichenoides (PL) chronica).

**Figure 1:**
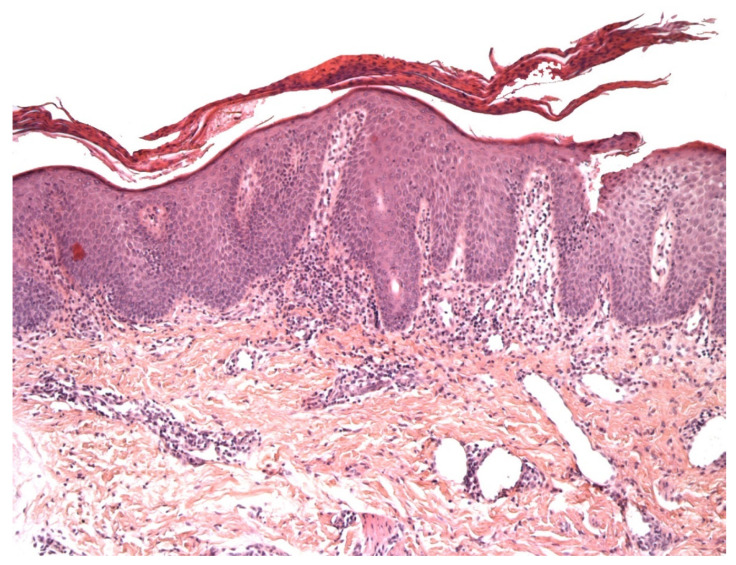
An example of vitiligo (inflammatory phase): parakeratosis, epidermal hyperplasia, slight spongiosis, lymphocyte exocytosis, and a discrete lymphocytic infiltrate in the superficial dermis. HE ×200.

In the early (inflammatory) phase of vitiligo, a marked superficial perivascular lymphocytic infiltrate can be observed; it sometimes has a lichenoid pattern and can be mistaken for epidermotropism [5] (Figure 1). Moreover, the intra-epidermal lymphocytes in inflammatory vitiligo are predominantly CD8-positive [6], as in hypopigmented MF [4,6]. Furthermore, the melanocyte count can also be abnormally low in cases of hypopigmented MF [6]. On one hand, some features tend to indicate a diagnosis of hypopigmented MF: partial depigmentation, the persistence of some melanocytes, wiry fibrosis of the papillary dermis, and increased density of the dermal infiltrate. On the other hand, some features argue in favor of vitiligo: complete loss of pigmentation, the complete absence of melanocytes, and fewer lymphocytes in the papillary dermis. When observed, the loss of cell surface “pan-T” antigens (CD2, CD5, and CD7) and the presence of clonal T-cell receptor rearrangements may be of diagnostic value. In difficult cases, the observation of clinical-pathological correlations and the course of the disease should enable a definitive diagnosis.

### 2.2. Immunodeficiencies Rashes

**Figure 2:**
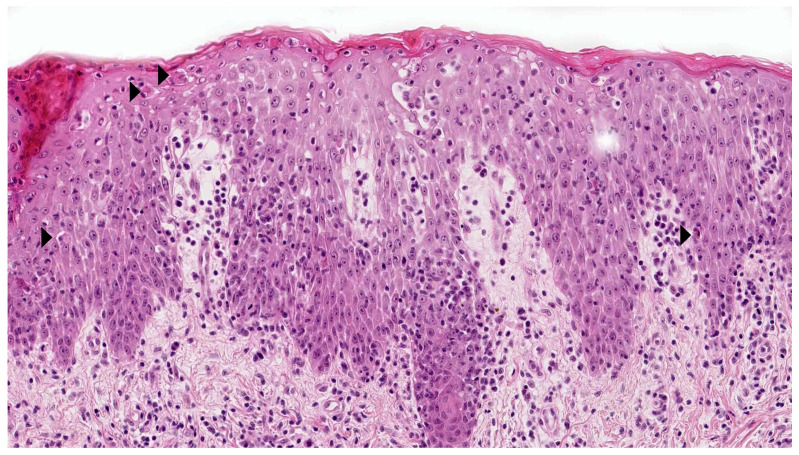
IPEX syndrome: Epidermal hyperplasia, marked lymphocytic exocytosis, scattered apoptotic keratinocytes (arrowhead), and a moderate lymphocytic infiltrate in the superficial dermis. HE ×400.

A skin rash (sometimes presenting as neonatal erythroderma) is a feature of several immunodeficiencies, including severe combined immunodeficiency, Omenn syndrome, and immune dysregulation-poly-endocrinopathy-enteropathy-X-linked syndrome [7]. These histological features can mimic MF; in particular, marked lymphocytic exocytosis resembles the epidermotropism seen in MF. This epidermotropism is commonly associated with adnexotropism. To the best of our knowledge, however, neonatal MF has never been described. However, the presence of necrotic/apoptotic changes in the epidermis and adnexal epithelium in a newborn/infant skin biopsy must alert the clinician and prompt him or her to consider a diagnosis of immunodeficiency (Figure 2).

### 2.3. Pityriasis Lichenoides

**Figure 3:**
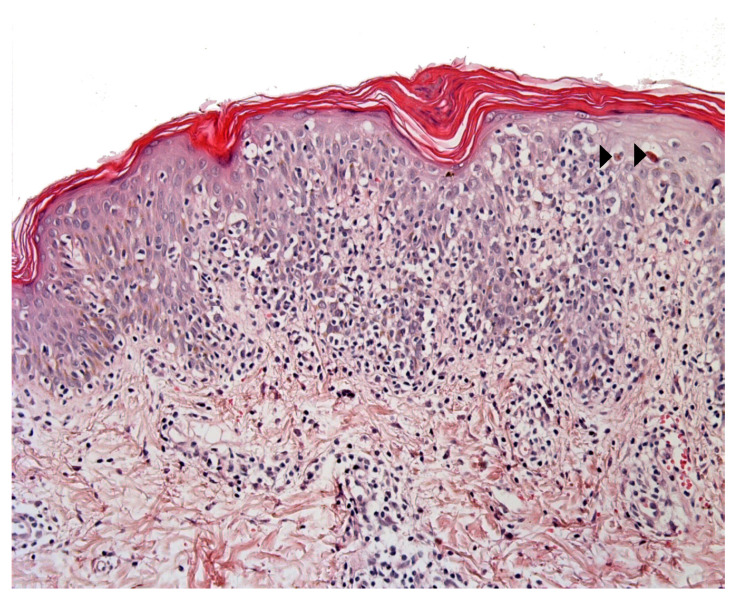
Mycosis fungoides-like pityriasis lichenoides. In this example there is a marked lymphocytic exocytosis, with lymphocytes displaying a clear halo. Note the few necrotic keratinocytes (arrowhead). HE ×250.

Pityriasis lichenoides (PL) is an infrequent skin disorder that predominantly affects children and young adults. The clinical presentation usually differs from that of MF, although the histological picture can be very similar. Moreover, PL can be present before (or at the same time as) MF in children. The histological picture in PL usually includes parakeratosis, epidermal hyperplasia, variable numbers of necrotic keratinocytes, interface changes with prominent lymphocytic exocytosis, perivascular, and periadnexal lymphocytic infiltrates in the superficial and deep dermis, and red blood cell extravasation [8]. However, some cases present with prominent intra-epidermal lymphocytes, basal cell replacement by lymphocytes, and nuclei surrounded by a clear halo—as in MF (Figure 3). The immunohistochemical findings are not usually helpful, unless “pan-T” antigen (CD2, CD5, and CD7) loss is highlighted. Moreover, a screen for monoclonality is not very helpful because many PL cases show a clonal T-cell receptor rearrangement [9,10]. Some findings tend to indicate a diagnosis of PL: the presence of necrotic keratinocytes, superficial epidermal pallor, red blood cell extravasation (especially intra-epidermal extravasation), and the presence of deep perivascular and periadnexal lymphocytic infiltrates.

Some cases of PL are difficult to distinguish clinically from lymphomatoid papulosis (LyP); this is notably the case when the lesions are necrotic and few in number. Moreover, large CD30+ lymphoid cells can be observed in the infiltrate in the epidermis, the dermis or both in some cases of PL, which can cause confusion with LyP [11]. Furthermore, monoclonality is not very helpful for differentiating between the two entities because many PL cases show a clonal T-cell receptor rearrangement [9,10]. However, some findings tend to indicate a diagnosis of PL: an intra-epidermal lymphocytic infiltrate associated with necrotic keratinocytes, cellular monomorphism with a complete absence of eosinophils, and few or no large lymphoid cells. Furthermore, our recent study showed that the lymphocytic infiltrate is usually wedge-shaped in LyP (except for the recently described follicular variant) but is T-shaped and follows the adnexa in PL [8].

### 2.4. Scabies Nodules and Insect Bite Reactions

The histological features of insect bite reactions (including scabies and post-scabies nodules) may mimic those of LyP, with a dense, lymphoid, wedge-shaped infiltrate containing large numbers of eosinophils and atypical or large CD30+ lymphoid cells [12,13]. The presence of CD30+ large lymphoid cells is a feature of many infectious diseases of the skin and is considered to be a sign of lymphocyte activation [14]. CD30 is a member of the tumor necrosis factor super-family and probably has a role in the immune response to infections [15]. In LyP in children, the high eosinophil count can mask the presence of large, atypical lymphocytes. A screen for monoclonality may help in some cases. Clinical-histological correlations, and sometimes additional biopsies or response to empiric therapy, are often the only means of making a firm diagnosis [16].

### 2.5. Lymphoplasmacytic Plaque

**Figure 4:**
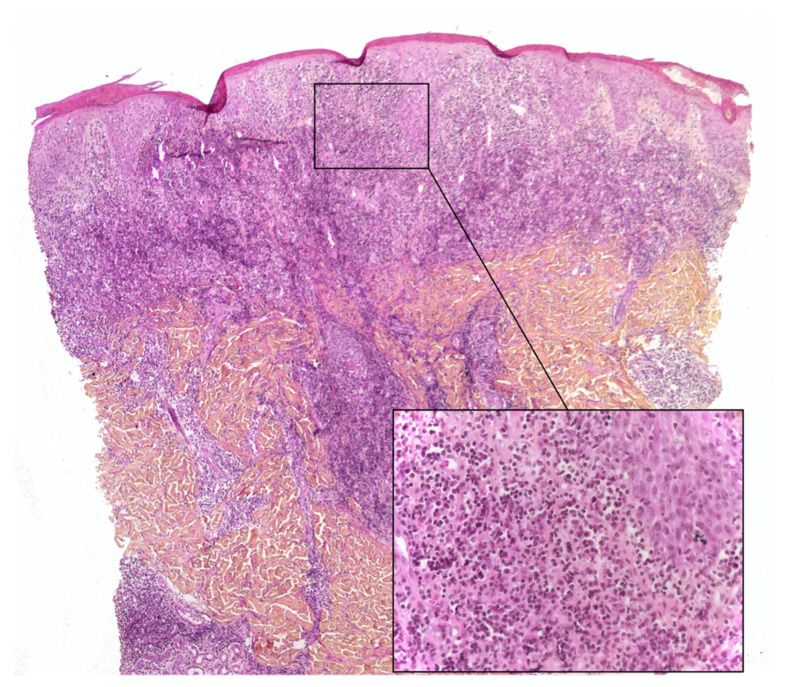
Lymphoplasmacytic plaque: parakeratotic scale, epidermal hyperplasia, a dense lymphoplasmacytic infiltrate in the whole dermis. Inset: note the numerous plasma cells. HE ×40 (inset: ×200).

Lymphoplasmacytic plaque (LPP) is a rare, recent characterized clinicopathological entity that mostly affects children. It features an asymptomatic, linear, reddish-brown, violaceous plaque, usually on the leg—hence the name “tibial” or “pretibial” LPP [17,18]. Since the initial reports, however, cases affecting other parts of the body have been described [19]. The histopathology is characterized by a dense nodular infiltrate within the upper reticular dermis or the whole dermis. This infiltrate is an admixture of plasma cells, lymphocytes, scattered histiocytes and (in some cases) epithelioid granulomas, together with vascular hyperplasia. The overlying epidermis may be hyperplastic and spongiotic [17,20] (Figure 4). This disease is closely related to another pseudo-lymphomatous disorder called “acral pseudolymphomatous angiokeratoma of children” (APACHE, see below) and may be a part of the same spectrum [20]. Importantly, the histological presentation of LP can be confused with that of cutaneous marginal zone lymphoma, a low-grade B cell lymphoma that very rarely affects children and occasionally affects teenagers [21]. Immunohistochemical analysis of the plasma cells in LPP shows a polytypic pattern of immunoglobulin light chain expression, and PCR studies do not show monoclonality [18,20,22].

### 2.6. Acral Pseudo-Lymphomatous Angiokeratoma of Children

Acral pseudo-lymphomatous angiokeratoma of children (APACHE), also named papular angiolymphoid hyperplasia, is a rare disease with female predominance [23]. It is characterized clinically by unilateral, asymptomatic, erythematous-violaceous papules and nodules with an acral distribution [24]. The histology findings are characterized by a dense superficial and deep dermal infiltrate, and a prominent vascular pattern of capillaries lined with plump endothelial cells. The infiltrate comprises lymphocytes, histiocytes, plasma cells, and eosinophils. The epidermis may also show hyperkeratosis, parakeratosis, spongiosis, and lymphocyte exocytosis [24]. Immunohistochemical studies show a slight predominance of T lymphocytes (with equal numbers of CD4+ and CD8+ cells, or slightly more CD4+ cells) and a large number of B lymphocytes. To the best of our knowledge, the presence or absence of CD30 in APACHE has not been studied immunohistochemically. A PCR analysis reveals that APACHE is a polyclonal disorder [25,26].

## 3. Histiocytic Infiltrates

### 3.1. CD1a+ Dendritic Cell Hyperplasia

CD1a+ dendritic cell hyperplasia (CD1a+ DCH) has been described in children as part of a large number of disorders including scabies, arthropod bite reactions, prurigo, warts, molluscum contagiosum, spongiotic dermatoses, psoriasis, PL, and interface dermatitis [27,28,29]. CD1a+ DCH can also be observed in proliferative processes, such as regressive melanocytic nevi, lymphoproliferative disorders, and the stroma of various tumors [30,31]. The CD1a+ DCH may have the typical aspect of Langerhans cell histiocytosis (LCH), i.e., round, medium-sized histiocytes with eosinophilic cytoplasm and, in some cases, large reniform or “coffee bean” shaped nuclei. This LCH-mimicking phenomenon is often referred to as “Langerhans cell hyperplasia” and can lead to misdiagnosis. Some cases do not display these characteristic morphologic features and are only discovered after immunostaining with anti-CD1a [28,32]. The Langerhans cells are often located in the dermis around the vessels, but can also be encountered in the epidermis—mostly in spongiotic disorders, such as Langerhans cell microgranulomas and pseudo-Pautrier abscesses [32]. There are a few histological features that facilitate the differential diagnosis between CD1a+ DCH and LCH:

- The infiltrate’s architecture: in CD1a+ DCH, cells are mostly located around the vessels in the dermis and do not accumulate in the papillary dermis as in LCH;
- The infiltrate’s polymorphism: the CD1a+ cells are rarely predominant and are mixed with other cell types;
- Immunoreactivity: importantly, the cells are not true Langerhans cells because they do not usually express CD207 (Langerin); in fact, they are CD1a+/CD207− dendritic cells that lack Birbeck granules [27,33]. CD207 is a very sensitive, specific marker of Birbeck granules, which are located in the cytoplasm of Langerhans cells. Along with CD207, the presence of markers of MAPK pathway activation (involved in the pathogenesis of LCH [34]) can also help the pathologist to distinguish between CD1a+ DCH and LCH. In particular, cyclin D1 and pERK are expressed in all cases of LCH but are not expressed (or only weakly) in cases of “dermatitis” with CD1a+ DCH [35]. The CD1a+/CD207− dendritic cells probably come from bone marrow (as do Langerhans cells and interdigitated DCs) and might be indeterminate cells or immature Langerhans cell precursors.

**Figure 5:**
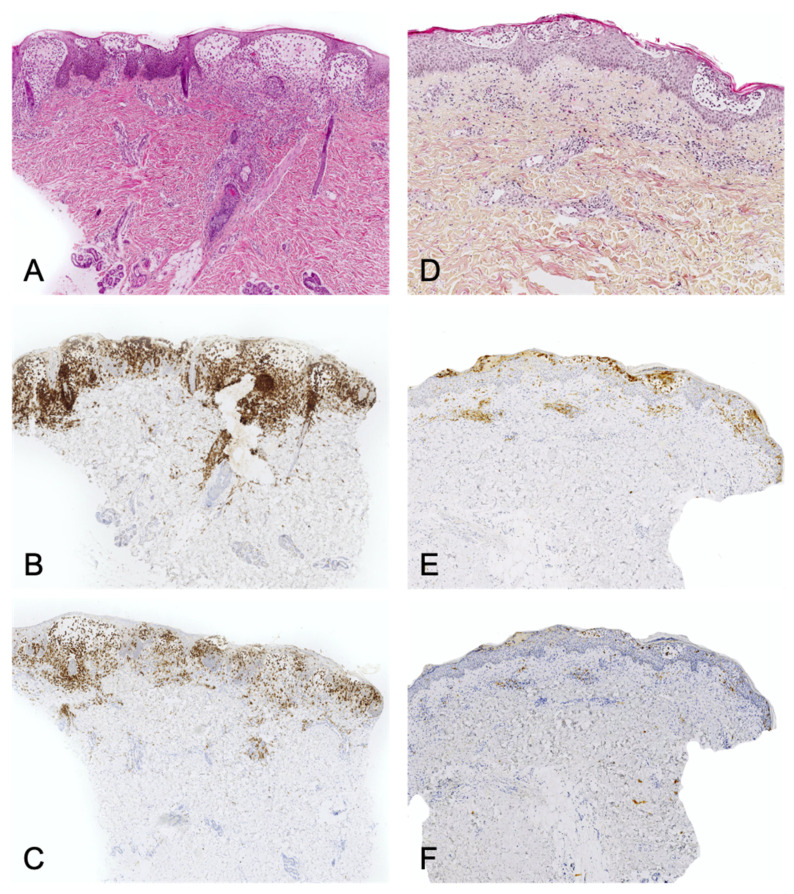
Left panel: Langerhans cell histiocytosis. (**A**): HE ×40. Superficial infiltrate of histiocytes with epidermotropism and edema. (**B**): CD1a immunostaining. (**C**): CD207/Langerin immunostaining. The whole infiltrate express CD207. Right panel: spongiotic dermatitis with CD1a+ dendritic cell hyperplasia. (**D**): HE ×40. (**E**): CD1a immunostaining. (**F**): CD207/Langerin immunostaining. The true Langerhans cells in the epidermal vesicles express CD207. The dermal infiltrate is almost completely negative.

Hence, immunostaining with antibodies against CD207 (and perhaps pERK and cyclin-D1) is very helpful for the differential diagnosis of CD1a+ DCH vs. LCH. In all cases, a clinical–histological correlation is always required for a firm diagnosis (Figure 5).

### 3.2. Juvenile Xanthogranuloma

Juvenile xanthogranuloma (JXG) is the most frequent subtype of non-Langerhans cell histiocytosis and one of the commonest skin tumors in children. This benign disorder usually affects young children and typically presents as a single erythematous to yellow papule, nodule, or plaque in the head and neck area. JXG usually regresses spontaneously and is very rarely associated with systemic disease [36]. Most JXG lesions appear during the first few years of life. There are several variants: micronodular, macronodular, giant (>5 cm), and subcutaneous JXG are observed. The giant and subcutaneous types always occur in the neonatal period. Clinically, JXG may be misleading and worrisome because of its size; it can mimic sarcoma in general and dermatofibrosarcoma protuberans in particular. Accordingly, a biopsy is essential.

These neonatal cases of JXG can also mimic malignancies histologically, since they usually display a large number of mitotic figures and have a high mitotic index (Ki67). Furthermore, early-stage JXG does not show xanthomization, and the cells tend to be monomorphous or even spindled. Early-stage JXG may also lack granulomatous inflammatory cells and, thus, can mimic spindle-cell sarcoma, round-cell sarcoma, or monoblastic leukemia. However, the cells do not show marked atypia and are commonly CD163+ and FXIIIa+—at least focally.

## 4. Melanocytic Disorders

Melanoma is rare in children; it accounts for 3% of all pediatric cancers. Both sexes are affected equally. Although 2% of melanomas occur in patients under the age of 20, only 0.3% occur in prepubertal children. The most common types are Spitz melanomas in prepubescent children and “conventional” melanomas (i.e., similar to chronic sun-damage melanomas seen in adults) in postpubescent children [37]. Some melanomas are associated with a congenital nevus; this can often be large and occasionally develops even before birth. However, most pediatric melanomas develop *de novo* in the absence of a known underlying condition. A single large (≥20 cm) congenital nevus or two or more congenital nevi (regardless of the size), abnormal central nervous system MRI findings in the first months of life are factors that predispose to congenital nevi-associated melanoma. Conventional melanoma is linked to genetic factors, such as a light complexion, poor tanning ability, sun exposure, xeroderma pigmentosum, and germline mutations of the *CDKN2A* gene [38,39].

A number of common but histologically ambiguous melanocytic lesions can easily be misconstrued as melanoma; they include (i) Spitz nevus and its variants, (ii) proliferative nodules in congenital nevi, (iii) acquired melanocytic nevi on particular, “special” anatomic sites (such as the scalp, genital area, acral sites, and conjunctiva), and (iv) lesions on the blue nevus spectrum [40].

### 4.1. Melanocytic Lesions in Newborns

Melanoma is very rare in the neonatal period, and there are very few case reports in the literature. The tumor either arises in a giant congenital melanocytic nevus (CMN) or grows from transplacental metastases of the mother’s melanoma [38,41]. The estimated risk of developing a melanoma associated with CMN is between 1% and 2% but might be as high as 10% to 15% for large and giant CMN. Early-onset (congenital, neonatal, and infantile) melanoma usually has a poor prognosis [41]. Melanocytic lesions that arise in association with a congenital melanocytic nevus will be discussed in the next subsection. 

**Figure 6:**
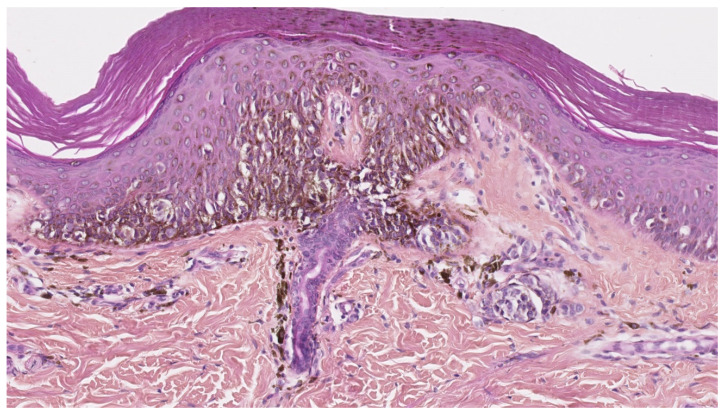
Junctional component of a congenital nevus in a newborn. Irregularly distributed melanocytes with pagetoid spread. HE ×250.

Melanocytic lesions in newborns can show worrisome histological features. The junctional component may show irregularly distributed and sometimes non cohesive nests. Pagetoid spread (a helpful criterion in the diagnosis of adult melanoma) should be interpreted with caution because it is displayed by many melanocytic lesions in newborns [40] and can, therefore, mimic superficial spreading melanoma (SSM) (Figure 6). The lesions may also be nodular and ulcerated, with many mitotic figures and a high proliferation index (Ki67/MIB); it can, therefore, mimic nodular melanoma. These lesions must be interpreted carefully so that melanoma is not erroneously diagnosed in this population. Ancillary immunochemistry or molecular biology test can be helpful (see the following section). Ideally, the clinician should seek an expert opinion before diagnosing melanoma. If doubt persists, excision of the lesion with broad margins (if possible) and long-term follow-up is recommended.

### 4.2. Melanocytic Lesions Associated with a Congenital Melanocytic Nevus

A distinct, benign, nodular proliferation arising on a congenital melanocytic nevus (CMN) and particularly on a giant CMN is referred to as a “proliferative nodule” (PN). This entity is relatively common and can mimic the clinical and histological features of melanoma [42]. The cell density is higher in the PN than in the adjacent congenital nevus. A PN can sometimes exhibit worrisome clinical features, such as rapid growth or ulceration. The typical histological characteristics of melanoma can also be seen in PN; these include a high mitotic index, sheets of large melanocytes with an epithelioid morphology, nuclear atypia, and even atypical mitoses and necrosis [43]. The features of this kind of lesion must be interpreted with caution because large/giant CMN is nevertheless the main risk factor for the development of melanoma in childhood [44,45].

**Figure 7:**
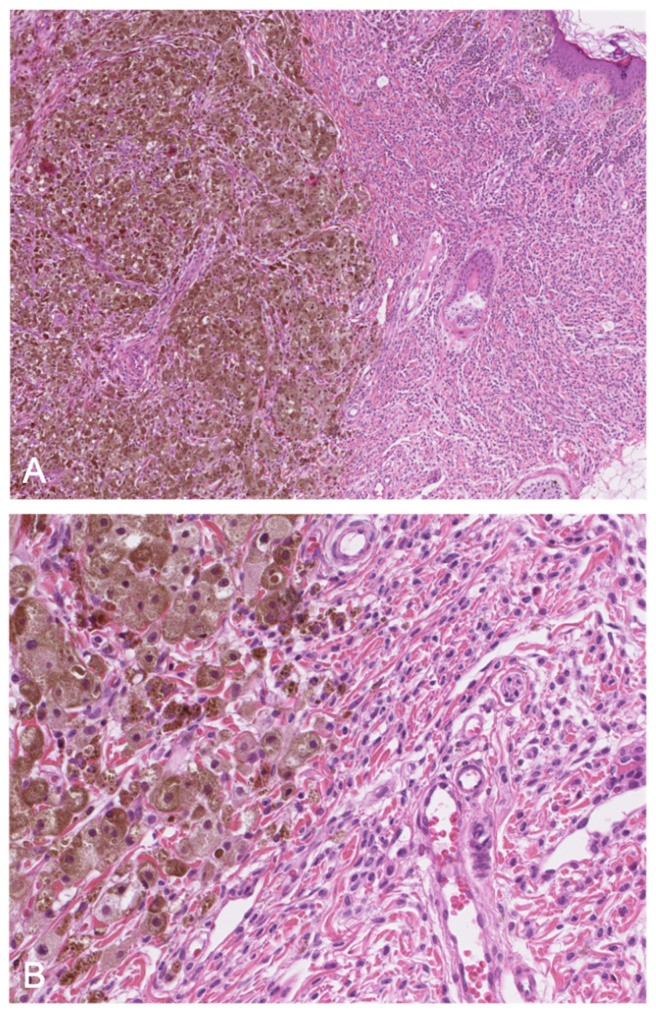
(**A**): (HE ×40) Proliferative nodule in a congenital melanocytic lesion. (**B**): (HE ×250) The melanocytes of the proliferative nodule are epithelioid and with a dusty cytoplasm, without nuclear atypia. Note the delicate transition (blending), which may be very focal and inconspicuous.

Several subtypes or morphological patterns have been described, including epithelioid, Spitzoid, small round blue cell-like, blue nevus-like, nevoid melanoma-like, and complex subtypes [43]. The recent literature has provided many characteristics, features, and clues of value in distinguishing between melanoma and PN [43,46,47]. The first important observation is that PN is much more frequent than melanoma. Secondly, and in contrast to melanoma, PN presents frequently as multiple lesions. Ulceration is suggestive of melanoma, especially when extensive. Blending (i.e., a smooth transition between PN cells and nevus cells), favors PN, even when focal (Figure 7). However, this feature is not always observed, and its significance is subject to debate. A very high mitotic index, necrosis within the nodule, inflammation, pleomorphism, and high-grade atypia increase the likelihood of a diagnosis of melanoma [41].

Immunochemistry is often described as being of limited value in distinguishing between PN and melanoma [48,49]. Recently, the expression of 5-hydroxymethylcytosine (5-hmC, an intermediate product of DNA demethylation) was studied in cases of giant CMN, PN, and melanoma arising within a CMN. Strong nuclear staining for 5-hmC was observed in almost all benign giant CMNs (93%) and PNs (98%) but never in melanomas associated with a within a CMN [42]. Immunochemical screening for the trimethylated lysine residue of histone H3 (H3K27me3) is also of diagnostic value: H3K27me3 expression tends to be abnormally low most nodular melanomas associated with a giant CMN [40,50].

Furthermore, molecular assays (such as fluorescence in situ hybridization (FISH) and comparative genomic hybridization (CGH)) have been studied in PN and melanoma arising in a CMN. FISH with standard melanoma probes was not helpful in distinguishing between PN and childhood-onset melanoma in a small series of patients [49]. In contrast, CGH might be useful for distinguishing between PN and melanoma because whole-chromosome gains or losses are observed in PNs, whereas and gains or losses in melanoma are restricted to parts or fragments of chromosomes [40,51]. An in situ hybridization study of telomerase reverse transcriptase mRNA gave promising results, with signals in all melanomas arising in a giant CMN but none in PNs [52]. 

### 4.3. Juvenile Nevi

**Figure 8:**
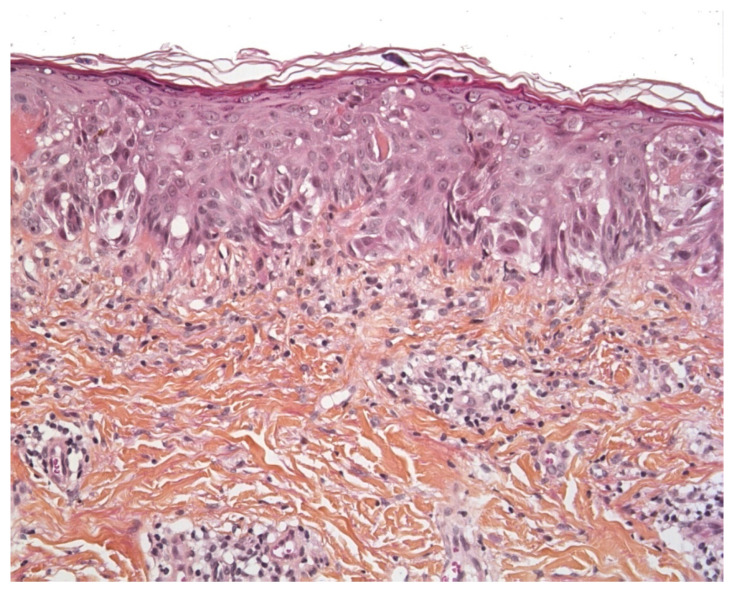
Pagetoid Spitz nevus (HE ×250). Epithelioid or spindle melanocytes with large nuclei and abundant “ground glass” cytoplasm, and pagetoid spread.

In principle, SSM does not occur in prepubertal children. However, common, acquired “juvenile-type” nevi occasionally show worrisome histological features that mimic SSM. Most of these lesions present as epithelioid Spitz nevi with a predominantly epithelioid appearance characterized by enlarged round-to-oval nuclei, often with a delicate open chromatin pattern. Some lesions are flat but some may be polypoid. These “juvenile-type” nevi may contain suprabasal intraepidermal melanocytes—especially in young children. At most, one can see features of “pagetoid Spitz nevus”, with the prominent pagetoid growth of solitary units and nests of melanocytes in the stratum spinosum throughout the lesion (Figure 8). Care must be taken not to confuse a pagetoid Spitz nevus with in situ melanoma. The presence of Spitzoid cytologic features, a small lesion diameter, and sharp demarcations are important parameters for the diagnosis of a Spitz nevus rather than SSM [53]. 

### 4.4. Spitz Nevus and Atypical Spitz Tumor

Spitz nevus and Spitzoid melanocytic lesions are part of a spectrum of melanocytic proliferations with a distinctive cellular morphology. The clinical-pathologic classification, diagnosis, and management of these lesions are among the most problematic topics in dermatopathology [54].

Histologically, Spitz nevi are mainly compound nevi and consist of epithelioid and spindle melanocytes with large nuclei and abundant “ground glass” cytoplasm. They show symmetry, sharp demarcation, uniform maturation (zonation), epidermal hyperplasia, Kamino bodies, no pleomorphism, no high-grade atypia, few mitoses, no mitoses in deep tissue, and no subcutaneous tissue involvement. 

Some Spitzoid lesions present atypia and are, therefore, referred to as an “atypical Spitz nevi” or “atypical Spitz tumors”. These atypia include a diameter >10 mm, asymmetry, subcutaneous fat involvement, a “pushing” deep margin, ulceration, poor demarcation, pagetoid migration, lack of maturation and zonation, few or no Kamino bodies, a high mitotic index (>2–6/mm^2^), deep mitoses, a proliferation index ≥ 10%, and cytological atypia (e.g., a high nucleocytoplasmic ratio, hyperchromatism, and large nucleoli) [55,56]. Spitzoid lesions are discussed more exhaustively elsewhere in this issue.

## 5. Conclusions

Many skin conditions in children can mimic the clinical and histologic features of malignancies. The pathologist must bear in mind that a neoplasm in a young child (and especially a newborn) will frequently have a high mitosis count or mitotic index, which are usually indicative of malignancy in adults. All dermatologists and dermatopathologists are aware of the importance of clinical–pathological correlations. This is particularly true in pediatrics, and these correlations—along with careful monitoring of the disease’s clinical course—sometimes constitute the only means of reaching a firm diagnosis. Except during the neonatal period, cutaneous malignancies are very rare in children; hence, the clinician is more likely to make a false-positive diagnosis than to truly miss a malignant disorder. It is also very important to ask for a second opinion from an expert center, when indicated.
